# Immune Thrombocytopenic Purpura (ITP) Following Natural COVID-19 Infection

**DOI:** 10.7759/cureus.26582

**Published:** 2022-07-05

**Authors:** Sisira Santhosh, Bilal Malik, Atefeh Kalantary, Arvind Kunadi

**Affiliations:** 1 Internal Medicine, Adichunchanagiri Institute of Medical Sciences, Rajiv Gandhi University of Health Sciences, B.G. Nagara, IND; 2 Internal Medicine, McLaren Health Care/Michigan State University (MSU), Flint, USA; 3 Internal Medicine/Nephrology, McLaren Health Care/Michigan State University (MSU), Flint, USA

**Keywords:** covid-19, covid-19 vaccine, itp management, adverse effects of covid, immune thrombocytopenia (itp)

## Abstract

Immune thrombocytopenic purpura (ITP) has been linked to the COVID-19 vaccine series as a rare adverse event but has recently emerged in the literature as a sequela of natural COVID-19 infection. ITP is a diagnosis of exclusion where a diagnosis is made by having isolated thrombocytopenia (platelet count <100,000/μL) and no other identifiable etiology for the thrombocytopenia. We share the case of a young male without any history of hematological or immunological disorders presenting with severe, symptomatic thrombocytopenia following a natural COVID-19 infection. Patients should be made aware of the potential risk of adverse events with not only vaccination but also even mild cases of natural infection with COVID-19. An emphasis should be placed on the fact that the benefits of vaccination continue to outweigh the potential risks of adverse events, even in those with a pre-existing diagnosis of ITP.

## Introduction

As of February 21, 2022, there were over 425 million global cases of coronavirus disease 2019 (COVID-19). Though COVID-19 has been most known for its effects on the respiratory system, namely severe acute respiratory distress syndrome, it has been increasingly identified as having multi-system implications [1]. These implications have been identified as occurring both acutely and chronically, sometimes long after the initial viral infection has resolved. Complications have ranged from renal failure to coagulopathies. Several unique hematological conditions have been identified in patients who have COVID-19 but also in those receiving specific vaccinations against the virus. Examples of such conditions include immune thrombocytopenic purpura (ITP), autoimmune hemolytic anemia (AIHA), and various cell line depressions.

Immune thrombocytopenic purpura (ITP) was initially identified more commonly with COVID-19 vaccines but has since become evident in patients with natural COVID-19 infection as well. We present the case of a young Caucasian male with a past medical history of hemochromatosis, type 2 diabetes mellitus, and two prior COVID-19 infections, who presented with epistaxis, hematuria, and petechial rash on his skin. His labs were significant for a platelet count of 0 k/μL. He was extensively worked up for hematologic etiologies, but ultimately none were identified. He was treated for ITP secondary to COVID-19 infection and successfully recovered. At present, increasing data is becoming available on ITP in COVID-19 infection, however, subjects developing a platelet count of 0 k/μL is rare.

## Case presentation

We present the case of a 23-year-old Caucasian male with a past medical history significant for hemochromatosis, type 2 diabetes mellitus, and two prior COVID-19 infections. He presented to the emergency department with lower extremity purpura, refractory epistaxis, and hematuria. His last COVID-19 infection was three months prior to presentation. On initial evaluation, his vitals included a heart rate (HR) of 65 beats per minute (bpm), respiratory rate of 14 breaths per minute, blood pressure of 143/95mmHg, and a pulse oximetry reading of 97%. A physical examination of the cardiovascular, respiratory, abdominal, and genitourinary systems was unremarkable. Of note, the patient did have widespread petechial rash across his lower limbs and crusted blood in his nares, bilaterally. Mucosal membranes demonstrated no apparent bleeding, aside from the above-documented epistaxis. His labs were only significant for thrombocytopenia, with no evidence of hemolytic anemia or derangement of liver function tests (Table 1). Abdominal ultrasound was performed and unrevealing.

**Table 1:** Summary of lab workup performed to rule out confounding conditions CBC: complete blood count; WBC: white blood cell count; RBC: red blood cell count; Hgb: hemoglobin; MCV: mean cell volume; MCHC: mean cell hemoglobin concentration; MCH: mean cell hemoglobin; PTT: partial thromboplastin time; INR: international normalized ratio; ANA: anti-nuclear antibody screen; IgA, M, G: immunoglobulin A, M, G

Lab parameter	Timeline			
CBC	February 18, 2022	February 19, 2022	February 20, 2022	February 21, 2022
WBC	8.29k/µL	7.47-7.97k/µL	5.47k/µL	5.32k/µL
RBC	6.07 million/µL	5.47million/µL	4.44 million/µL	4.01 million/µL
Hb	17g/dL	15.6g/dL	12.4g/dL	11.5g/dL
Platelets	3000/µL	0/µL	6000/µL	41000/µL
MCV	82fL	82.1-82.4fL	83fL	85fL
MCHC	34.1g/dL	34.4g/dL	33.3g/dL	33.7g/dL
MCH	28pg	28.3-28.5pg	27.9pg	28.7pg
RBC morphology	-	-	Normal	Normal
Platelet sufficiency	Significantly decreased	Significantly decreased	Significantly decreased	Significantly decreased
Hemoglobin A1C	-	-	10.4	-
Partial thromboplastin time	36.5	29.2-34.9	-	-
Thrombin clotting time	-	16.9	-	-
Immediate PTT 1:1 MX	-	26.2	-	-
Prothrombin time	-	10.6	-	-
INR	-	0.96	-	-
LA ratio	-	1.81	-	-
LA ratio mix	-	1.42	-	-
Hexagonal phospholipid neutralisation	-	31.0	-	-
ANA screen	-	Negative	-	-
Beta 2 glycoprotein IgA, IgG, IgM	-	Negative	-	-
Cardiolipin IgG, IgM, IgA	-	Negative	-	-
COVID-19	-	Negative	-	-

On review of systems, he denied any flu-like symptoms, fever, chills, abdominal pain, nausea, vomiting, diarrhea, or bloody stools. His family history was significant for hemochromatosis and diabetes mellitus in both his mother and grandmother. He denied any family history of bleeding disorders but did report that his mother had delayed healing with injuries. He denied using any smoking products, alcohol, or recreational drugs. His only home medication was insulin and he denied any recent changes to his regimen. The patient was initially admitted to the wards and was in stable condition. He received 1 unit of platelets in view of thrombocytopenia, with a platelet count of 3000/μL, and concurrent bleeding. Following platelet transfusion, his thrombocytopenia progressed, with his platelet count dropping to 0 /μL. Due to his progressive thrombocytopenia and symptomatic state, the patient was transferred to the ICU for closer monitoring. He received four additional units of platelets with no improvement in his thrombocytopenia.

Based on the extensive studies performed, it was concluded that the patient had viral immune-mediated thrombocytopenia - a diagnosis of exclusion (as outlined in Table 1). Prednisone 100mg daily and two doses of intravenous immunoglobulin (IVIG) (at a dose of 1g/kg) were administered. He received treatment for two days and his platelet counts improved significantly (Table 1). Once stabilized, he was discharged on a course of prednisone 50mg, twice daily, for outpatient follow-up with his primary provider and hematology. 

## Discussion

Clinical manifestations of COVID-19 can range from asymptomatic clinical course to acute respiratory distress depending on viral load, host immunity, and existing comorbidities. Hematological changes have been documented to varying degrees, depending on the patient's immune response and the severity of the infection.

Immune thrombocytopenic purpura is caused by antibodies against platelet glycoprotein 2b/3a complex. The three diagnostic criteria for immune thrombocytopenia include (1) Isolated thrombocytopenia with otherwise normal peripheral blood smear and count; (2) absence of hepatosplenomegaly, and lymphadenopathy on physical examination; and (3) platelet response to classic ITP therapy - steroids, IVIG, anti-D [2].

Epidemiology

ITP is a commonly acquired bleeding disorder. The reported incidence appeared to increase with the introduction of automated platelet counting in the 1970s; however, this was likely due to an increase in the incidental finding of thrombocytopenia rather than a true increase in the incidence of ITP [3-10]. The annual ITP incidence is estimated at one to six per 100,000 adults in the era of routine complete blood counts; this estimate is based on three large retrospective studies from Europe and a study from Korea [3-10]. ITP is often a chronic disease in adults; thus its prevalence tends to exceed its incidence. In a review from the United States, the prevalence was approximately eight per 100,000 in children and 12 per 100,000 in adults [3-10].

Other studies have estimated that one-fifth to one-third of individuals with ITP will be asymptomatic at the time of diagnosis (i.e., they are diagnosed on incidental lab findings of thrombocytopenia); thus, the incidence of symptomatic disease is likely to be much lower [3-10]. In a database review from the French National Health Insurance System that was limited to ITP cases requiring chronic therapy and/or hospitalization, the overall incidence was 2.9 per 100,000 person-years, with a peak in individuals older than 60 years of age, reaching nine cases per 100,000 person-years.

Diagnosis

ITP is a diagnosis of exclusion. It is defined as isolated thrombocytopenia (platelet <100,000/μL) without anemia or leukopenia, and no other identifiable etiology for the thrombocytopenia. A presumptive diagnosis of primary ITP can be made when history, physical examination, and laboratory testing (including review of the peripheral smear) do not reveal other potential etiologies for thrombocytopenia. A presumptive diagnosis of secondary ITP can be made in a patient with ITP and an underlying associated condition (such as anti-phospholipid syndrome, systemic lupus erythematosus, etc.) [2].

Management

The goals of treatment should be to treat or prevent significant bleeding. Platelet count normalization should not be pursued. In patients with critical bleeding causing hemodynamic or respiratory compromise, platelet transfusion is used in all patients. Glucocorticoids and IVIG are used rather than either therapy alone. For those with severe bleeding (Hb decrease 2g/dL or requiring > 2 units transfusion), glucocorticoids alone are preferred rather than IVIG alone or in combination with glucocorticoids. Those with minor bleeding and severe thrombocytopenia without bleeding should be managed with an individualized treatment plan (minor bleeding/platelet count <20,000, treatment is preferred over observation) [10].

Anti-D immune globulin is periodically used as an alternative to conventional IVIG for patients whose blood type is RhD-positive, but many clinicians are hesitant to use it because of the risk of severe hemolytic transfusion reactions, for which there is a food and drug administration (FDA) boxed warning [8,11].

Multiagent combinations, second-line agents like thrombopoietin receptor agonists (TPO-RAs), rituximab, and splenectomy are reserved for patients who fail first-line therapy. Individuals with active ITP should have platelet counts monitored before and after COVID-19 vaccination. It is important to note that the benefits of vaccination outweigh the risk of an exacerbation in the majority of patients. Table 2 summarizes other cases of ITP, their presentation, subsequent management, and vaccination status (if available). 

**Table 2:** Summary of several cases of ITP after natural COVID-19 infection, illustrating possible presentation, disease course, and interventions made by various authors. IVIG: intravenous immunoglobulin; COVID-19: coronavirus disease 2019; ITP: immune thrombocytopenic purpura; Y: year

S. no.	Case	Patient demographics	Presentation	Platelet level trend (per microliter)	Intervention	Vaccination
1	Zulfiqar et al. (2020) [12]	65Y female	4 days of fever, fatigue, dry cough, abdominal discomfort. Covid 19 positive	183,000 >16,000 > 8000 >on treatment >10,000 > 139,000	Platelet transfusion, prednisolone 100mg, eltrombopag 75mg/day	Not reported
2	Malik et al. (2020) [13]	29Y female	Malaise, body ache. COVID-19-positive	20,000 > 18,000 > 7000 >on treatment >12,000 > 16,000 >24,000 > 166,000	4 units of platelets, prednisolone 1mg/kg	Not reported
3	Bennett et al. (2020) [14]	73Y female	Fever, shortness of breath, diarrhea, fatigue, body ache, cough. Covid 19 positive	<3000 (undetectable platelets) > on treatment> 105,000 > 146,000	1 unit apheresis platelets, IVIG 1g/kg/day - 2 doses, hydroxychloroquine 400mg	Not reported
4	Sadr et al. (2020) [15]	57Y female	Headache, malaise. Covid 19 positive	48,000 >19,000 on treatment 26,000 > 4000 >144,000		Not reported
5	Nham et al. (2020) [16]	54Y male	Headache, productive cough, myalgia, fever, epistaxis, petechiae.	154,000 >135,000 >113,000 >2000	Lopinavir, levofloxacin, ceftriaxone	Not reported
6	Lorenzo-Villalba et al. (2020) [17]	57Y female	Fever, dry cough, shortness of breath, epistaxis, cutaneous purpura	2000> on treatment> 75,000	IVIG, eltrombopag	Not reported
7	Lorenzo-Villalba et al. (2020) [17]	66Y male	Fever, cough, shortness of breath, diarrhea, epistaxis	73,000 > 1000>on treatment>20,000 > 149,000	IVIG, eltrombopag	Not reported
8	Levraut et al. (2021) [18]	63Y female	Fever, dry cough, headache	197,000 >3000 >on treatment>38,000 > 95,000 > 145,000	IVIG	Not reported

## Conclusions

Here, we presented the case of a young adult male with no previously diagnosed hematological or immunological disorders who presented with severe thrombocytopenia following a natural COVID-19 infection. All hematological workup was negative for any preexisting coagulopathies and by the definition the patient has ITP. The patient received IVIG and a high dose of steroids with subsequent improvement in his thrombocytopenia. The current literature has documented ITP cases following vaccination for COVID-19 abundantly, but there is limited literature concerning ITP following natural COVID infection. This case highlights the importance of educating the public regarding possible risks associated with not only COVID vaccination but also with COVID infection itself. It also highlights the importance of emphasizing to the patient population that the benefits outweigh the risks with regard to vaccination, even in patients with known histories of ITP, in the vast majority.

## References

[1] (2022). Number of cumulative cases of coronavirus (COVID-19) worldwide from January 22, 2020 to June 26, 2022, by day. https://www.statista.com/statistics/1103040/cumulative-coronavirus-covid19-cases-number-worldwide-by-day/.

[2] Cines DB, Blanchette VS (2002). Immune thrombocytopenic purpura. N Engl J Med.

[3] Terrell DR, Beebe LA, Vesely SK, Neas BR, Segal JB, George JN (2010). The incidence of immune thrombocytopenic purpura in children and adults: a critical review of published reports. Am J Hematol.

[4] Frederiksen H, Schmidt K (1999). The incidence of idiopathic thrombocytopenic purpura in adults increases with age. Blood.

[5] Abrahamson PE, Hall SA, Feudjo-Tepie M, Mitrani-Gold FS, Logie J (2009). The incidence of idiopathic thrombocytopenic purpura among adults: a population-based study and literature review. Eur J Haematol.

[6] Terrell DR, Beebe LA, Neas BR, Vesely SK, Segal JB, George JN (2012). Prevalence of primary immune thrombocytopenia in Oklahoma. Am J Hematol.

[7] Weycker D, Grossman A, Hanau A (2018). Immune thrombocytopenia in US clinical practice: incidence and healthcare utilization/expenditures in the first 12 months following diagnosis. Blood.

[8] Tarantino MD, Bussel JB, Cines DB (2007). A closer look at intravascular hemolysis (IVH) following intravenous anti-D for immune thrombocytopenic purpura (ITP). Blood.

[9] Weycker D, Hanau A, Hatfield M (2020). Primary immune thrombocytopenia in US clinical practice: incidence and healthcare burden in first 12 months following diagnosis. J Med Econ.

[10] Neunert C, Terrell DR, Arnold DM (2019). American Society of Hematology 2019 guidelines for immune thrombocytopenia. Blood Adv.

[11] Gaines AR (2007). Response: further examination of intravascular hemolysis (IVH) following intravenous anti-D for immune thrombocytopenic purpura (ITP). Blood.

[12] Zulfiqar AA, Lorenzo-Villalba N, Hassler P, Andrès E (2020). Immune thrombocytopenic purpura in a patient with COVID-19. N Engl J Med.

[13] Malik J, Javaid M, Majedi O, Ishaq U, Zahid T (2020). Paying in blood: a case of thrombocytopenia in COVID-19. Cureus.

[14] Bennett J, Brown C, Rouse M, Hoffmann M, Ye Z (2020). Immune thrombocytopenia purpura secondary to COVID-19. Cureus.

[15] Sadr S, SeyedAlinaghi S, Ghiasvand F, Hassan Nezhad M, Javadian N, Hossienzade R, Jafari F (2020). Isolated severe thrombocytopenia in a patient with COVID-19: a case report. IDCases.

[16] Nham E, Ko JH, Jeong BH (2020). Severe thrombocytopenia in a patient with COVID-19. Infect Chemother.

[17] Lorenzo-Villalba N, Zulfiqar AA, Auburtin M (2020). Thrombocytopenia in the course of COVID-19 infection. Eur J Case Rep Intern Med.

[18] Levraut M, Ottavi M, Lechtman S, Mondain V, Jeandel PY (2021). Immune thrombocytopenic purpura after COVID-19 infection. Int J Lab Hematol.

